# LOw-Dose RAbeprazole Therapy for Reducing Gastrointestinal Events in Patients with High Bleeding Risk (LORA-HBR): A Prospective, Multicenter, Interventional Study

**DOI:** 10.3390/jcm15031289

**Published:** 2026-02-05

**Authors:** Dong Oh Kang, Cheol Ung Choi, Jang Hoon Lee, Young Joon Hong, Jung-Sun Kim, Han Cheol Lee, Jay Young Rhew, Jang Hyun Cho, Weon Kim

**Affiliations:** 1 Cardiovascular Center, Department of Internal Medicine, Korea University Guro Hospital, Korea University College of Medicine, Seoul 08308, Republic of Korea; gelly9@naver.com (D.O.K.); wmagpie@korea.ac.kr (C.U.C.); 2 Division of Cardiology, Kyungpook National University Hospital, Kyungpook National University School of Medicine, Daegu 41944, Republic of Korea; ljhmh75@knu.ac.kr; 3 Department of Cardiology, Chonnam National University Hospital, Chonnam National University Medical School, Gwangju 61469, Republic of Korea; hyj200@hanmail.net; 4 Division of Cardiology, Severance Hospital, Yonsei University College of Medicine, Seoul 03722, Republic of Korea; kjs1218@yuhs.ac; 5 Division of Cardiology, Pusan National University Hospital, Pusan National University School of Medicine, Pusan 49241, Republic of Korea; glaraone@hanmail.net; 6 Division of Cardiology, Presbyterian Medical Center, Jeonju 54987, Republic of Korea; jyrhew@nate.com; 7 Division of Cardiology, St. Carollo Hospital, Suncheon 57931, Republic of Korea; goodnew8@naver.com; 8 Department of Cardiology, Kyung Hee University Hospital, Kyung Hee University College of Medicine, Seoul 02447, Republic of Korea

**Keywords:** anticoagulants, cardiovascular disease, dual antiplatelet therapy, gastrointestinal hemorrhage, proton pump inhibitors, rabeprazole

## Abstract

**Background:** The widespread use of antithrombotic therapies increases bleeding risk, particularly in patients with a high bleeding risk (HBR). Although proton pump inhibitors are recommended for lowering the risk of upper gastrointestinal (UGI) bleeding, the optimal agent and dosage remain uncertain. This study evaluated the efficacy and safety of low-dose rabeprazole (LORA, 5 mg) in reducing the incidence of GI-related adverse events in HBR patients receiving chronic antithrombotic therapy. **Methods:** This was a prospective, multicenter, interventional study that enrolled 909 South Korean patients receiving long-term antithrombotic therapy with HBR features including age ≥70 years, dual antiplatelet therapy, combined antithrombotic regimens, and prior GI bleeding. The primary endpoint was the incidence of significant GI events, including overt/occult bleeding and symptomatic peptic ulcer disease (PUD). Secondary endpoints included study drug discontinuation owing to GI adverse events, composite cardiovascular events, and all-cause mortality. **Results:** No patients had significant UGI bleeding or symptomatic PUD. The median adherence rate was 92.0% (interquartile range [IQR], 87.0–95.0). Drug discontinuation owing to GI symptoms occurred in 32 patients (3.52%) at a median of 81 days (IQR, 36–119 days). GI-related adverse events were reported in 3.96%, with diarrhea, epigastric discomfort, and constipation being the most common. Non-GI bleeding and cardiovascular composite events occurred in 0.33% (*n* = 3) each, with all-cause mortality at 0.55% (*n* = 5). **Conclusions:** Low-dose rabeprazole was associated with reduced GI complications in patients receiving chronic antithrombotic therapy, with a favorable safety profile and high adherence. Further studies with larger and broader populations are required to confirm these findings.

## 1. Introduction

The growing prevalence of cardiovascular diseases (CVDs) has led to a significant increase in the use of antithrombotic therapies, which has raised concerns about the associated risk of bleeding events [1,2]. Patients receiving prolonged dual antiplatelet therapy (DAPT) or combined antithrombotic regimens are particularly vulnerable to these complications [3,4,5]. Among individuals treated with coronary drug-eluting stents, bleeding events, especially gastrointestinal (GI) bleeding, remain a major concern [6]. These bleeding events have been shown to have an equal or greater impact on all-cause mortality compared to post-discharge myocardial infarction (MI) [6,7]. To mitigate this risk, current guidelines recommend prophylactic proton pump inhibitor (PPI) therapy for high-risk patients on DAPT, oral anticoagulants (OAC), or combined antithrombotic regimens [8,9,10,11].

Although the efficacy of PPIs in reducing upper GI (UGI) bleeding is well-established across various clinical settings [12], the optimal agent and dosage for patients receiving antithrombotic therapy remain uncertain. Among the available PPIs, rabeprazole is distinguished by its unique pharmacokinetic and pharmacodynamic properties, including rapid onset of action, potent acid suppression, and minimal dependence on cytochrome P450 2C19 (CYP2C19) metabolism [13]. Owing to these features, rabeprazole is particularly beneficial for patients with genetic variations in CYP2C19 or those on medications that compete for this metabolic pathway. Low-dose rabeprazole (5 mg) has been shown to be effective in preventing UGI adverse events in patients on low-dose aspirin therapy with a history of peptic ulcer disease (PUD) [14]. Moreover, rabeprazole has minimal interference with the antiplatelet effects of clopidogrel, making it a safer option for patients requiring both PPI and antithrombotic therapy [15]. Despite these advantages, the efficacy of low-dose rabeprazole in reducing GI adverse events (AEs) in high-risk patients on chronic antithrombotic therapy has not been thoroughly explored. To address this issue, the present study aimed to evaluate the efficacy and safety of low-dose rabeprazole in reducing adverse GI events in patients with high bleeding risk (HBR) receiving antithrombotic therapy.

## 2. Materials and Methods

### 2.1. Study Design and Population

Low-dose Rabeprazole Therapy for Reducing Gastrointestinal Events in Patients with High Bleeding Risk (LORA-HBR) was a prospective, multicenter, interventional study conducted from January 2022 to January 2024 across seven cardiology referral centers in South Korea. Patient enrollment was conducted between January and September 2022, aiming to include individuals requiring at least one year of long-term antithrombotic therapy who exhibited one or more HBR characteristics. These included age ≥70 years, use of multiple or high-dose nonsteroidal anti-inflammatory drugs (NSAIDs), DAPT, combined antithrombotic therapy with OAC and antiplatelet agents, history of PUD or GI bleeding, and systemic steroid therapy. The exclusion criteria were active bleeding or hemodynamic instability at enrollment; hereditary or acquired hemostatic disorders; hypersensitivity or contraindication to PPIs; concurrent use of strong CYP3A4 and P-gp inhibitors; treatment with atazanavir, nelfinavir, or rilpivirine-containing medications; pregnancy or breastfeeding; life expectancy of <12 months; significant laboratory abnormalities at screening; history of venous disorder or non-endoscopic gastric surgery; recent fibrinolytic therapy; suspected malignancy; or contraindications to antithrombotic therapy. The study protocol was approved by the Institutional Review Board (IRB) of each participating center and was conducted in accordance with the Declaration of Helsinki. Written informed consent was obtained from all participants before enrollment.

### 2.2. Patient Management and Assessment

At enrollment, baseline demographic information, physical examination findings, medical history, current prescriptions, and laboratory test results were recorded. For patients with a history of percutaneous coronary intervention (PCI), the timing of the most recent revascularization procedure was also documented. Laboratory data included tests performed within four weeks prior to enrollment. The patients were instructed to take a daily dose of 5 mg rabeprazole (Pariet^®^, rabeprazole sodium, Eisai Co., Seoul, Republic of Korea) at the same time each day. This selected dose was based on prior evidence demonstrating its preventive efficacy against aspirin-induced UGI AEs [14]. Follow-up assessments were scheduled at 6- and 12-month post-enrollment through outpatient clinic visits, with additional visits conducted at the discretion of the attending clinician. Drug adherence was assessed at each outpatient visit by calculating the difference between the total number of pills dispensed and the number remaining divided by the number of days since enrollment. In addition to prespecified clinical HBR criteria, individual bleeding risk was quantitatively assessed using validated scoring systems that could be calculated from the available clinical variables, including the Patterns of Non-Adherence to Anti-Platelet Regimen in Stented Patients (PARIS) score and Coronary Revascularization Demonstrating Outcome Study in Kyoto (CREDO-Kyoto) bleeding risk score [16,17].

### 2.3. Study Endpoints

The primary endpoint was the cumulative incidence of composite clinical events originating from GI sources, with the definition modified from previous research [18,19]. This included overt UGI bleeding confirmed by esophagogastroduodenoscopy (EGD) or abdominal computed tomography (CT); obvious UGI bleeding of an unknown origin; bleeding of presumed occult GI origin leading to significant blood loss, defined as a hemoglobin decrease ≥2 g/dL or hematocrit drop ≥10% from baseline; symptomatic PUD confirmed by EGD or abdominal CT; and symptomatic gastric or duodenal erosion affecting more than five locations, confirmed by EGD, with symptom persisting more than three days. Secondary endpoints included time to development of primary composite endpoints; time to study drug discontinuation due to GI symptoms; incidence of non-GI bleeding events; occurrence of erosive esophagitis or gastroesophageal reflux disease (GERD) confirmed by EGD or abdominal CT; cardiovascular events, defined as a composite of cardiac death, nonfatal MI, and nonfatal stroke; individual components of cardiovascular events; all-cause mortality; and potential side effects associated with rabeprazole administration.

The safety endpoints included any AEs requiring clinical attention during the study drug administration period. AEs were classified as those leading to the discontinuation of the study drug and those specifically associated with GI symptoms. Adverse GI events included constipation, nausea/vomiting, diarrhea, abdominal discomfort, epigastric soreness, isolated nausea, dyspepsia, anorexia, loose stools, and minor GI bleeding. Non-GI AEs included allergic reactions, fever, sore throat, hand and foot edema, arthralgia, genitourinary symptoms, malignancy, nonspecific fatigue, dyspnea, skin abnormalities, dizziness, chest pain, coronary revascularization, headache, and other symptoms or hospital admissions not otherwise classified.

### 2.4. Statistical Analysis

The sample size calculation was based on a previously reported 1.5% risk of GI bleeding events associated with long-term antithrombotic therapy and an odds ratio of 0.37 for the protective effect of PPI therapy [20,21]. Given an estimated event rate of 0.56%, the required sample size was calculated to be 958 patients. To accommodate a potential 5% dropout rate, the target enrollment was set at 1000 patients. Data are presented as mean ± standard deviation (SD) or median with interquartile range (IQR) for continuous variables and as frequency (percentage) for categorical variables. The cumulative incidence of the outcome variable of interest is displayed using a survival curve. Statistical significance was defined as a *p*-value <0.05. All analyses were two-tailed. Analyses were performed using the Statistical Package for the Social Sciences (SPSS) software (version 20.0, SPSS-PC Inc., Chicago, IL, USA).

## 3. Results

### 3.1. Patient Characteristics

**Figure 1:**
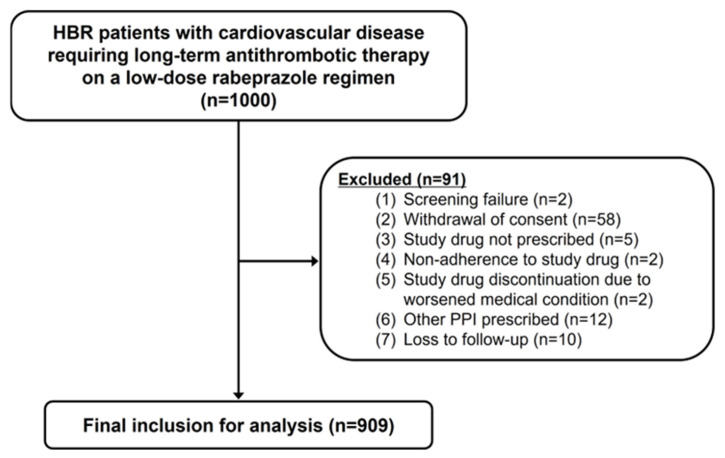
Flowchart of study enrollment. Abbreviations: HBR, high bleeding risk; PPI, proton pump inhibitor.

Figure 1 shows a flowchart of the study enrollment. Of the 1000 patients initially enrolled, 909 were included in the final analysis after applying the exclusion criteria. The baseline demographic characteristics are summarized in Table 1. The mean age of participants was 72.9 ± 8.3 years, with 71.3% (*n* = 648) being male. The average follow-up period was 364 days (IQR, 350–385 days). Most patients presented with one (74.4%, *n* = 676) or two (24.6%, *n* = 224) HBR characteristics, with the most common factor being age ≥70 years (75.7%, *n* = 688), DAPT (41.9%, *n* = 381), and combined antithrombotic therapy with OAC and antiplatelet agents (4.2%, *n* = 38). Comorbidities included hypertension in 75.2% (*n* = 684), diabetes in 35.8% (*n* = 325), and dyslipidemia in 55.7% (*n* = 506) of patients. A history of coronary artery disease (CAD) was present in 93.7% (*n* = 852) of the cohort, with 57.6% (*n* = 524) having experienced acute coronary syndrome. Among CAD patients, the majority had undergone PCI (86.1%; *n* = 783), with an average time from the index procedure of median 4.36 (IQR, 1.61–8.71) years. Bleeding risk stratification using the PARIS score identified 43.1% (*n* = 392) and 10.1% (*n* = 92) of patients as intermediate and high risk, respectively, while the CREDO-Kyoto score classified 38.9% (*n* = 354) and 7.8% (*n* = 71) of patients into the corresponding risk categories. The baseline laboratory findings are presented in Table 2.

### 3.2. Patient Management and Medications

Table 3 summarizes prescribed medications. All patients received 5 mg of low-dose rabeprazole daily, with a median adherence rate of 92.0% (IQR, 87.0–95.0). The adherence rates of ≥70% and ≥80% were achieved by 93.8% (*n* = 853) and 88.7% (*n* = 806) of patients, respectively. Clopidogrel was the most common antiplatelet therapy (72.3%, *n* = 657), followed by aspirin (61.2%, *n* = 556), cilostazol (2.3%, *n* = 21), and prasugrel (1.9%, *n* = 17). Among the 11.9% (*n* = 108) of patients receiving anticoagulants, direct oral anticoagulants (DOACs) were predominant (96.2%, *n* = 104), and warfarin was prescribed at the lowest rate (3.8%, *n* = 4). Reduced-dose DOACs were more frequently prescribed (71.2%, *n* = 74) than standard-dose regimens (28.8%, *n* = 30), reflecting the HBR features of the cohort. Concomitant NSAIDs were prescribed to 1.1% (*n* = 10) of patients, while non-PPI GI-protective agents were prescribed to 2.0% (*n* = 18) of patients.

### 3.3. Primary and Secondary Outcomes

**Figure 2:**
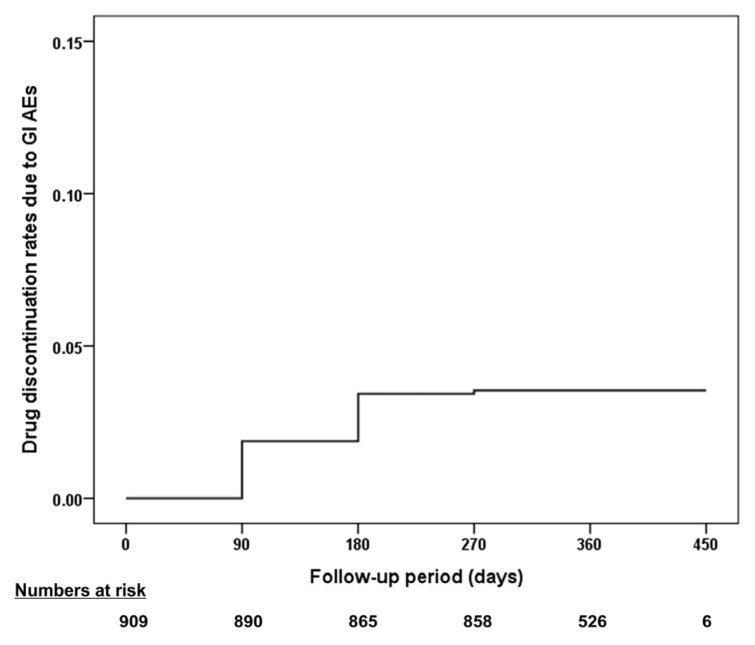
Survival curve of study drug discontinuation due to GI AEs. Abbreviations: AE, adverse event; GI, gastrointestinal.

Table 4 presents the primary and secondary outcomes. None of the patients experienced the primary composite endpoints of significant UGI bleeding or symptomatic PUD while receiving low-dose rabeprazole. Study drug discontinuation owing to GI symptoms was reported in 32 patients (3.52%) at a median of 81 days after enrollment (IQR, 36–119 days; Figure 2). One patient (0.11%) developed symptomatic gastric erosion as confirmed by EGD. Non-GI bleeding events were observed in three patients (0.33%). For cardiovascular safety, three prespecified events (0.33%) were recorded, including one case each of cardiovascular death, nonfatal MI, and nonfatal stroke. All-cause mortality occurred in five patients (0.55%). Exploratory outcomes revealed minor GI bleeding in one patient (0.11%) and coronary revascularization in six patients (0.66%).

### 3.4. Safety Outcomes of AEs

Table 5 summarizes the AEs observed in this study. A total of 99 AEs were reported in 86 patients (9.46% of the study population). GI-related AEs occurred in 36 patients (3.96%), with diarrhea and abdominal pain (0.99%, *n* = 9), epigastric discomfort (0.99%, *n* = 9), and constipation (0.99%, *n* = 9) being the most common symptoms (Table 6). Non-GI AEs were reported in 52 patients (5.72%), including chest pain (1.21%, *n* = 11), hospitalization owing to underlying conditions (0.88%, *n* = 8), allergic skin reactions (0.66%, *n* = 6), dizziness (0.66%, *n* = 6), and coronary revascularization (0.66%, *n* = 6) (Table 7). Study drug discontinuation owing to AEs occurred in 36 (3.96%) patients. Of these, 88.9% (*n* = 32) were GI-related AEs and 11.1% (*n* = 4) were non-GI AEs.

## 4. Discussion

In this prospective multicenter interventional study, we found that low-dose rabeprazole (5 mg) was well tolerated by HBR patients receiving chronic antithrombotic therapy, with a median adherence rate of 92.0%. Notably, no patients experienced significant UGI bleeding or symptomatic PUD, which was confirmed by EGD during the study period. GI AEs were reported in 3.96% of patients and primarily included mild symptoms such as diarrhea, abdominal pain, epigastric discomfort, and constipation. The rate of study drug discontinuation owing to GI-related AEs was 3.52%, with a median time to discontinuation of 81 days. Cardiovascular safety was also maintained with a very low incidence of cardiovascular mortality, nonfatal MI, and nonfatal stroke. Collectively, these findings suggest that low-dose rabeprazole may be an effective and generally well-tolerated option for reducing GI complications in high-risk patients receiving chronic antithrombotic therapy, with an acceptable risk–benefit profile.

### 4.1. UGI Bleeding Risk with DAPT and the Role of PPI Prophylaxis

Our study demonstrated an exceptionally low rate of UGI bleeding in patients with HBR who received chronic antithrombotic therapy with low-dose rabeprazole. In this cohort, the most common HBR characteristics were advanced age ≥70 years (75.7%), DAPT use (41.9%), and combined antithrombotic therapy (4.2%). DAPT is known to cause more significant gastric mucosal injury than single antiplatelet therapy, thus increasing the risk of clinically relevant bleeding events, particularly in HBR patients [22]. Previous randomized and real-world studies, most notably in the Clopidogrel and the Optimization of Gastrointestinal Events Trial (COGENT), have consistently shown that PPI co-therapy reduces UGI bleeding in patients treated with DAPT, supporting the protective role of PPIs in this population [18,23,24,25,26]. While direct quantitative comparisons with such landmark trials are limited by differences in study design, inclusion criteria, and baseline risk profiles, absence of significant UGI bleeding events in the present cohort is directionally consistent with the protective effect observed in prior studies. Importantly, when comparisons are restricted to PPI-treated cohorts, LORA-HBR differed from prior trials by including more elderly patients and those meeting clinical HBR criteria, yet demonstrated numerically lower GI event rates despite the use of a lower PPI dose. A descriptive comparison of PPI-treated populations across LORA-HBR and landmark studies, including COGENT, is summarized in Table 8, highlighting differences in patient characteristics, anti-thrombotic regimens, and observed GI outcomes. Collectively, these findings extend existing evidence by suggesting that effective GI protection may be achieved with a dose-optimized PPI strategy, such as low-dose rabeprazole, in selected HBR patients rather than routine use of higher-dose regimens.

### 4.2. UGI Bleeding Risk with OAC and Combined Antithrombotic Therapy

Patients on OAC or combined antithrombotic therapy are at an elevated risk of UGI bleeding, particularly in the presence of HBR characteristics [4,8,27]. In our study, 11.4% of patients (*n* = 104) received DOAC therapy, with the majority (71.1%, *n* = 74) prescribed reduced doses, reflecting real-world clinical decision-making in elderly and high-risk populations. In addition, 4.2% of patients (*n* = 38) were treated with combined antithrombotic therapy, further exemplifying the heightened baseline bleeding risk in our cohort. Despite these high-risk features, the incidence of UGI bleeding remained extremely low with low-dose rabeprazole therapy. Prior randomized trials and meta-analyses have demonstrated that PPI co-therapy reduces GI bleeding in patients receiving OAC or combination regimens, including evidence from the Cardiovascular Outcomes for People Using Anticoagulation Strategies (COMPASS) trial, supporting GI protection in patients treated with combined antithrombotic strategies [19,28,29,30]. However, direct extrapolation of event rates or hazard ratios to our study is inherently limited by differences in antithrombotic intensity, patient selection, and outcome definitions across trials. Rather than focusing on quantitative comparisons, our findings demonstrate that low-dose rabeprazole was associated with favorable GI outcomes even in patients receiving complex antithrombotic regimens in routine clinical practice. As summarized in Table 8, the LORA-HBR cohort represents a distinct real-world bleeding risk population, with broader inclusion of elderly and chronic phase patients receiving diverse antithrombotic regimens, and no clinically significant UGI bleeding events were observed. These results support the practical applicability of low-dose prophylaxis as an effective GI-protective strategy in HBR patients requiring long-term OAC or combined antithrombotic therapy.

### 4.3. Cardiovascular Risk of Concurrent PPI and Antithrombotic Therapy

Our study demonstrated a very low incidence of cardiovascular composite endpoints (0.33%, *n* = 3) among patients with HBR treated with low-dose rabeprazole and antithrombotic regimens, demonstrating the safety of this co-therapy. While concerns about potential interactions between PPIs and clopidogrel have been raised in the past [31], accumulating evidence has consistently shown that PPI co-therapy has a minimal impact on cardiovascular outcomes [18,30,32,33]. In the COGENT trial, no significant differences in the primary cardiovascular safety endpoint (cardiovascular death, nonfatal MI, coronary revascularization, or ischemic stroke) were observed between the omeprazole group (4.9%; 55/1876) and the placebo group (5.7%; 54/1885) at 180 days [18]. Similarly, the COMPASS trial found no significant differences in the incidence of primary cardiovascular outcomes (MI, stroke, or cardiovascular death) between PPI users (7.9%; 691/8791) and non-PPI users (7.6%; 668/8807), with a HR of 1.04 (95% CI 0.93–1.15) [32]. Meta-analyses provide further clarity on this issue. Studies including observational data have suggested an increased risk of MACE with PPI use; however, analyses limited to RCTs consistently showed no significant difference in MACE risk [26,33]. Similarly, meta-analyses of PPI co-therapy with combined antithrombotic therapy found no evidence of an increased risk of cardiovascular events associated with PPI use [30].

The cardiovascular risk in patients receiving antithrombotic therapy also depends on patient characteristics and treatment timing. In our study, most patients (86.1%) underwent PCI as an indication for antithrombotic therapy, with a median of 4.36 years since the most recent coronary intervention. It is well documented that thrombotic risk is highest during the early stage following PCI, whereas bleeding risk persists in the long term [34]. The low cardiovascular risk observed in our study likely reflects the stabilized phase of our patient population. These findings suggest that low-dose rabeprazole treatment in stabilized patients with HBR receiving antithrombotic therapy does not significantly increase cardiovascular risk, thus supporting its safety and clinical utility in this population.

### 4.4. Potential Advantages of Low-Dose Rabeprazole Therapy

To the best of our knowledge, our study provides the first real-world evidence of the efficacy and safety of low-dose rabeprazole in reducing GI complications in patients with HBR undergoing chronic antithrombotic therapy. This strategy addresses several challenges specific to high-risk populations and offers several distinct advantages. A primary concern with PPI and antiplatelet therapies, particularly clopidogrel, is the potential for drug–drug interactions. While in vitro and observational studies have raised this issue, RCTs have not identified a clinically significant risk [18,26,31,33]. Nevertheless, minimizing theoretical interaction risks remains prudent. The favorable metabolic profile of low-dose rabeprazole may alleviate such concerns by ensuring effective GI protection without substantially increasing the risk of adverse drug events [13]. This approach is especially relevant for East Asian populations, who exhibit lower thrombotic but higher bleeding risks compared to Western cohorts [35]. Such characteristics make prophylactic PPI therapy particularly beneficial. A low-dose regimen can expand PPI use while minimizing PPI-related AEs. Our study provides real-world evidence supporting the use of low-dose rabeprazole in East Asian patients, underscoring its value in addressing their unique risk–benefit considerations.

Additionally, prolonged and high-dose PPI use has been associated with adverse effects, including increased risks of cardiovascular events [36], GI infection [37], pneumonia [38], hip fracture [39], and kidney disease [40]. These risks are particularly concerning for vulnerable patients. A low-dose rabeprazole regimen may mitigate these complications because the likelihood of PPI-related risks tends to increase with extended use, higher doses, and preexisting comorbidities. By adopting a low-dose strategy, clinicians can balance effective GI protection with a reduced risk of AEs. Further supporting this approach, a recent Korean cohort study of DOAC users showed that PPI co-therapy, including low-dose regimens, effectively prevented UGI bleeding [41]. Although these findings require confirmation in RCTs, the observed protective effect of low-dose PPIs supports broader adoption, particularly in East Asian patients on DOAC therapy. By optimizing the dose and duration of PPI therapy, low-dose rabeprazole offers a practical, patient-oriented approach to minimize GI complications while reducing associated risks. Our findings reinforce the efficacy and safety of this approach in high-risk populations and provide valuable insights into the adaptation of this strategy to diverse patient groups with varying thrombotic and bleeding risk profiles.

### 4.5. Drug-Related AEs in Low-Dose Rabeprazole Therapy

Most patients tolerated low-dose rabeprazole without AEs, with AEs leading to drug discontinuation primarily related to GI issues. The median time to discontinuation was 81 days after therapy initiation, suggesting that factors unrelated to the study drug may have contributed to these GI-related AEs. Non-GI-related AEs such as chest pain or coronary revascularization were expected to be largely attributable to the underlying characteristics of patients with CAD rather than the study drug itself. Consequently, the rate of drug discontinuation owing to non-GI-related AEs was relatively low. These findings indicate that low-dose rabeprazole is generally well tolerated in patients with HBR, offering a favorable safety profile for reducing UGI events while maintaining good tolerability.

### 4.6. Limitations

This study had several limitations. First, as a single-arm interventional study without a control group, establishing a causal relationship between low-dose rabeprazole and the low incidence of GI bleeding events is inherently limited. The observed low event rate may have been influenced by the predefined clinical HBR characteristics and the natural clinical course, in addition to a potential contribution from low-dose rabeprazole. Including a control group without PPI therapy may enhance causal inference but raises ethical concerns given the elevated GI bleeding risk in high-risk patients. Second, the incidence of the primary outcome was lower than anticipated, and no GI bleeding events were recorded during the study period. Although this suggests that low-dose rabeprazole may effectively prevent GI bleeding in high-risk patients in a real-world setting, it also limits the statistical power of the study. Third, the relatively high rate of consent withdrawal may have introduced selection bias, as patients who withdrew could differ systematically from those who completed the study, potentially affecting the generalizability of our findings. Fourth, although all patients met prespecified clinical HBR inclusion criteria, a smaller proportion were classified as intermediate or high risk by the PARIS and CREDO-Kyoto bleeding risk scores. This discrepancy likely reflects the predominance of clinically stable, chronic phase patients in our cohort, characterized by a low prevalence of prior GI bleeding or PUD and a median of 4.36 years since index PCI. Accordingly, score-based bleeding risk estimates should be interpreted with caution in this setting, and this population profile may partly account for the low observed event rate. Fifth, while the concept of risk stratification for PPI use has been challenged [24], with some evidence supporting unguided PPI strategies to optimize bleeding risk in antithrombotic users, our approach in HBR patients offers flexibility and could be extended to broader populations, leveraging the benefits of a low-dose strategy. Finally, although a centralized adjudication committee was not used, this limitation is unlikely to have influenced the primary results and may potentially relate to minor GI events or study drug discontinuation.

## 5. Conclusions

The present study emphasized the clinical utility of low-dose rabeprazole in reducing the burden of GI complications without significantly increasing the risk of AEs, particularly in patients with HBR. Given its favorable safety profile and metabolic characteristics, low-dose rabeprazole is a viable option for prophylactic therapy in patients receiving long-term antithrombotic treatment. Future RCTs or large-scale studies are warranted to further validate these findings and explore the broader applicability of this approach in diverse clinical settings.

## Figures and Tables

**Table 1:** Baseline demographics of the study population.

	Study Population (*n* = 909)
Age (years)	72.90 ± 8.29
Sex (male, %)	648 (71.3)
Follow-up duration (days)	364.0 (350.0–385.0)
HBR characteristics	
Age ≥ 70 years (%)	688 (75.7)
Multiple or high-dose NSAIDs therapy (%)	11 (1.2)
Dual antiplatelet therapy (%)	381 (41.9)
Combined antithrombotic therapy ^a^ (%)	38 (4.2)
History of peptic ulcer disease (%)	19 (2.1)
History of GI bleeding (%)	8 (0.9)
Systemic steroid therapy (%)	6 (0.7)
Number of HBR characteristics	1.0 (1.0–2.0)
One (%)	676 (74.4)
Two (%)	224 (24.6)
Three or more (%)	9 (1.0)
Medical comorbidities	
Hypertension (%)	684 (75.2)
Diabetes mellitus (%)	325 (35.8)
Dyslipidemia (%)	506 (55.7)
Smoking (%)	84 (9.3)
Atrial fibrillation (%)	84 (9.2)
Cerebrovascular disease (%)	63 (6.9)
Peripheral arterial disease (%)	18 (2.0)
End-stage renal disease (%)	25 (2.8)
Previous CAD (%)	852 (93.7)
Myocardial infarction (%)	238 (26.2)
Unstable angina (%)	286 (31.5)
Stable angina (%)	295 (32.5)
Silent ischemia (%)	33 (3.6)
Previous revascularization therapy for CAD	
PCI (%)	783 (86.1)
CABG (%)	40 (4.4)
Timing of previous PCI (years)	4.36 (1.61–8.71)
Bleeding risk score	
PARIS bleeding score	4 (3–5)
Low risk (<4) (%)	425 (46.8)
Intermediate risk (4–7) (%)	392 (43.1)
High risk (>7) (%)	92 (10.1)
CREDO-Kyoto bleeding score	0 (0–1)
Low risk (0) (%)	484 (53.2)
Intermediate risk (1–2) (%)	354 (38.9)
High risk (>2) (%)	71 (7.81)

Data are presented as mean ± standard deviation, median (interquartile ranges), or frequency (percentage). ^a^ Combined antithrombotic therapy refers to the concurrent use of oral anticoagulants and antiplatelet agents. Abbreviations: CAD, coronary artery disease; CABG, coronary artery bypass graft surgery; CREDO, Coronary Revascularization Demonstrating Outcome Study; GI, gastrointestinal; HBR, high bleeding risk; PARIS, Patterns of Non-Adherence to Anti-Platelet Regimen in Stented Patients; PCI, percutaneous coronary intervention; NSAID, non-steroidal anti-inflammatory drug.

**Table 2:** Baseline laboratory findings.

	Study Population (*n* = 909)
Systolic blood pressure (mmHg)	127.43 ± 15.69
Diastolic blood pressure (mmHg)	69.89 ± 10.12
Heart rate (beats/min)	73.70 ± 11.27
Hemoglobin (g/dL)	13.54 ± 1.59
White blood cell count (×1000/μL)	6.41 ± 1.91
Platelet count (×1000/μL)	212.45 ± 56.40
AST (U/L)	28.36 ± 9.81
ALT (U/L)	26.31 ± 14.29
BUN (mg/dL)	18.51 ± 6.34
Serum creatinine (mg/dL)	0.91 ± 0.30
Prothrombin time (INR)	1.06 ± 0.35
Left ventricular ejection fraction (%)	61.01 ± 9.96

Data are expressed as mean ± standard deviation. Abbreviations: ALT, Alanine aminotransferase; AST, Aspartate aminotransferase; bpm, beats per minute; BUN, blood urea nitrogen; INR, international normalized ratio.

**Table 3:** Prescribed medications in the study population.

	Study Population (*n* = 909)
GI protective agents	
Low-dose rabeprazole of 5 mg (%)	909 (100.0)
Non-PPI GI-protective agents (%)	18 (2.0)
Adherence rate of low-dose rabeprazole (%)	92.0 (87.0–95.0)
≥70% (%)	853 (93.8)
≥80% (%)	806 (88.7)
≥90% (%)	557 (61.3)
Antiplatelet agents	
Dual antiplatelet therapy (%)	381 (41.9)
Aspirin (%)	556 (61.2)
Clopidogrel (%)	657 (72.3)
Prasugrel (%)	17 (1.9)
Ticagrelor (%)	7 (0.8)
Cilostazol (%)	21 (2.3)
Trifusal (%)	2 (0.2)
Sarpogrelate (%)	1 (0.1)
Oral anticoagulants	
Direct oral anticoagulants (%)	104 (11.4)
Standard-dose/Reduced-dose (%)	30 (3.3)/74 (8.1)
Rivaroxaban ^a^ (%)	41 (4.5)
Standard-dose/Reduced-dose (%)	7 (0.8)/34 (3.7)
Edoxaban ^b^ (%)	43 (4.7)
Standard-dose/Reduced-dose (%)	22 (2.4)/21 (2.3)
Apixaban ^c^ (%)	17 (1.9)
Standard-dose/Reduced-dose (%)	1 (0.1)/16 (1.8)
Dabigatran ^d^ (%)	3 (0.3)
Standard-dose/Reduced-dose (%)	0 (0.0)/3 (0.3)
Warfarin (%)	4 (0.4)
Combined antithrombotic therapy ^e^ (%)	38 (4.2)
NSAIDs (%)	11 (1.2)
Multiple NSAIDs (%)	1 (0.1)
Systemic steroid therapy (%)	6 (0.7)

Data are presented as median (interquartile ranges) or frequency (percentage). ^a^ Rivaroxaban: standard-dose (20 mg once daily) and reduced-dose (10 mg or 15 mg once daily). ^b^ Edoxaban: standard-dose (60 mg once daily) and reduced-dose (15 mg or 39 mg once daily). ^c^ Apixaban: standard-dose (5 mg twice daily) and reduced-dose (2.5 mg twice daily). ^d^ Dabigatran: standard-dose (150 mg twice daily) and reduced-dose (110 mg twice daily). ^e^ Combined antithrombotic therapy refers to the concurrent use of oral anticoagulants and antiplatelet agents. Abbreviations: GI, gastrointestinal; NSAID, non-steroidal anti-inflammatory drug; PPI, proton pump inhibitor.

**Table 4:** Primary and secondary study outcomes.

	Study Population (*n* = 909)
Primary GI composite outcome (%)	0 (0.0)
Overt UGI bleeding confirmed by EGD or CT (%)	0 (0.0)
Obvious UGI bleeding with an unknown origin (%)	0 (0.0)
Presumed occult GI bleeding with significant blood loss ^a^ (%)	0 (0.0)
Symptomatic PUD confirmed by EGD or CT (%)	0 (0.0)
Symptomatic gastric or duodenal erosion ^b^ (%)	0 (0.0)
Secondary GI outcome	
Study drug discontinuation due to GI symptoms (%)	32 (3.52)
Non-GI bleeding (%)	3 (0.33)
Erosive esophagitis or GERD confirmed by EGD or CT (%)	1 (0.11)
Minor GI bleeding ^c^ (%)	1 (0.11)
Secondary cardiovascular composite outcome ^d^ (%)	3 (0.33)
Cardiovascular death (%)	1 (0.11)
Nonfatal MI (%)	1 (0.11)
Nonfatal stroke (%)	1 (0.11)
All-cause death (%)	5 (0.55)
Coronary revascularization ^c^ (%)	6 (0.66)

Data are presented as frequency (percentage). ^a^ Significant blood loss is defined as a hemoglobin decrease ≥2 g/dL or hematocrit drop ≥10% from baseline. ^b^ Symptomatic gastric or duodenal erosion affecting more than five locations, confirmed by EGD, with symptoms persisting for more than three days. ^c^ Minor GI bleeding and coronary revascularization were assessed as exploratory outcomes. ^d^ The cardiovascular composite outcome includes cardiovascular death, nonfatal MI, and nonfatal stroke. Abbreviations: CT, computed tomography; EGD, esophagogastroduodenoscopy; GERD, gastroesophageal reflux disease; GI, gastrointestinal; MI, myocardial infarction; PUD, peptic ulcer disease; UGI, upper gastrointestinal.

**Table 5:** Adverse events and study drug discontinuation during the study period.

	Study Population (*n* = 909)
GI adverse events (%)	37 (4.07)
Non-GI adverse events (%)	52 (5.72)
Drug discontinuation due to adverse events (%)	36 (3.96)
Discontinuation due to GI adverse events (%)	32 (3.52)
Discontinuation due to non-GI adverse events (%)	4 (0.44)

Data are presented as frequency (percentage). Abbreviations: GI, gastrointestinal.

**Table 6:** Details of GI adverse events.

	Study Population (*n* = 909)
GI adverse events (%)	37 (4.07)
Constipation (%)	9 (0.99)
Nausea and vomiting (%)	4 (0.44)
Diarrhea and abdominal pain (%)	9 (0.99)
Epigastric soreness (%)	9 (0.99)
Abdominal discomfort (%)	6 (0.66)
Isolated nausea (%)	1 (0.11)
Dyspepsia (%)	4 (0.44)
Loose stool (%)	3 (0.33)
Anorexia (%)	1 (0.11)
Minor GI bleeding (%)	1 (0.11)

Data are presented as frequency (percentage). Abbreviations: GI, gastrointestinal.

**Table 7:** Details of Non-GI adverse events.

	Study Population (*n* = 909)
Non-GI adverse events (%)	52 (5.72)
Allergic reactions (%)	6 (0.66)
Fever or sore throat (%)	1 (0.11)
Hand or foot edema (%)	2 (0.22)
Arthralgia (%)	1 (0.11)
Genitourinary symptoms (%)	3 (0.33)
Malignancy diagnosis (%)	1 (0.11)
Nonspecific fatigue (%)	1 (0.11)
Dyspnea (%)	2 (0.22)
Skin abnormalities (%)	1 (0.11)
Dizziness (%)	6 (0.66)
Chest pain (%)	11 (1.21)
Coronary revascularization (%)	6 (0.66)
Headache (%)	1 (0.11)
Other symptoms not otherwise classified (%)	2 (0.22)
Hospital admissions not otherwise classified (%)	8 (0.88)

Data are presented as frequency (percentage). Abbreviations: GI, gastrointestinal.

**Table 8:** Comparison of PPI-treated cohorts across LORA-HBR and landmark trials.

	LORA-HBR	COGENT [18]	Danish RCT [24]	COMPASS [19]
PPI group/Total (%)	909/909 (100.0)	1876/3761 (49.9)	997/2009 (49.6)	8791/17,598 (50.0)
PPI drug used	Rabeprazole 5 mg	Omeprazole 20 mg	Pantoprazole 40 mg	Pantoprazole 40 mg
Age (years)	72.90 ± 8.29	68.5 (60.7–74.4)	64.7 ± 8.29	67.6 ± 8.1
Antithrombotic regimen				
DAPT (%)	381 (41.9)	1876 (100.0)	997 (100.0)	0 (0.0) ^b^
OAC (%)	108 (11.4)	0 (0.0) ^a^	49 (4.9)	5863 (66.7) ^b^
Antiplatelet + OAC (%)	38 (4.2)	0 (0.0) ^a^	49 (4.9)	2945 (33.5) ^b^
GI bleeding/ulcer history (%)	27 (3.0)	78 (4.2)	136 (13.6)	228 (2.6)
GI bleeding history (%)	19 (2.1)	n/a ^c^	108 (10.8)	0 (0.0) ^d^
GI ulcer history (%)	8 (0.9)	n/a ^c^	28 (2.8)	228 (2.6)
GI composite outcomes (%)	0 (0.0)	13 (0.7)	10 (1.0)	102 (1.2)
GI bleeding (%)	0 (0.0)	8 (0.4)	8 (0.8)	76 (0.9)
GI ulcer (%)	0 (0.0)	5 (0.3)	2 (0.2)	12 (0.1)

Data are expressed as frequency (percentage), mean ± standard deviation, or median (interquartile range). All comparisons are restricted to PPI-treated cohorts in each study to account for the 100% PPI use in the LORA-HBR study. ^a^ Patients receiving oral anticoagulation were excluded according to the original study design. ^b^ In the COMPASS trial, 2945 patients (33.5%) received rivaroxaban 2.5 mg twice daily plus aspirin 100 mg, while 2918 patients (33.2%) received rivaroxaban 5.0 mg twice daily. Patients treated with DAPT were excluded. ^c^ GI bleeding or ulcer history was reported as a composite variable. ^d^ Patients who had a high risk of bleeding were excluded according to the original study design. Abbreviations: COGENT, Clopidogrel and the Optimization of Gastrointestinal Events Trial; COMPASS, Cardiovascular Outcomes for People Using Anticoagulation Strategies; DAPT, dual antiplatelet therapy; GI, gastrointestinal; LORA-HBR, Low-dose Rabeprazole Therapy for Reducing Gastrointestinal Events in Patients with High Bleeding Risk; n/a, not available; OAC, oral anticoagulant; PPI, proton pump inhibitor; RCT, randomized controlled trial.

## Data Availability

The corresponding author (W.K.) had complete access to the study data and was responsible for the integrity and accuracy of the data analysis. Anonymized data can be obtainable upon reasonable request by qualified researchers.

## Abbreviations

The following abbreviations are used in this manuscript:

AE, adverse event; CT, computed tomography; CVD, cardiovascular disease; DAPT, dual antiplatelet therapy; EGD, esophagogastroduodenoscopy; GERD, gastroesophageal reflux disease; GI, gastrointestinal; HBR, high bleeding risk; IQR, interquartile range; MI, myocardial infarction; NSAID, nonsteroidal anti-inflammatory drug; OAC, oral anticoagulant; PCI, percutaneous coronary intervention; PPI, proton pump inhibitor; PUD, peptic ulcer disease; SD, standard deviation; UGI, upper gastrointestinal.
